# Reliability of anthropometric measures in a longitudinal cohort of patients initiating ART in West Africa

**DOI:** 10.1186/1471-2288-10-102

**Published:** 2010-10-22

**Authors:** Maryline Sicotte, Marielle Ledoux, Maria-Victoria Zunzunegui, Souleymane Ag Aboubacrine, Vinh-Kim Nguyen

**Affiliations:** 1 Département de Médecine Sociale et Préventive, Faculté de Médecine, Université de Montréal, Pavillon 1420 Mont Royal, C.P. 6128, succursale CV, Montréal, Québec, H3C 3J7, Canada; 2 Institut de Recherche en Santé Publique de l'Université de Montréal, Faculté de Médecine, Université de Montréal, Pavillon 1420 Mont Royal, C.P. 6128, succursale CV, Montréal, Québec, H3C 3J7, Canada; 3 Département de Nutrition, Pavillon Liliane-de-Stewart, Université de Montréal, C.P. 6128, succursale CV, Montréal, Québec, H3C 3J7, Canada; 4 Service de Médecine Interne, Hôpital National du Point G, BP 33, Bamako, Mali; 5 Axe de Recherche en Santé Mondiale, Centre de Recherche du Centre Hospitalier de l'Université de Montréal (CR-CHUM), 3875 rue Saint-Urbain, 5ième étage, Montréal, Québec, H2W 1V1, Canada

## Abstract

**Background:**

Anthropometric measurements are a non invasive, inexpensive, and suitable method for evaluating the nutritional status in population studies with relatively large sample sizes. However, anthropometric techniques are prone to errors that could arise, for example, from the inadequate training of personnel. Despite these concerns, anthropometrical measurement error is seldom assessed in cohort studies. We describe the reliability and challenges associated with measurement of longitudinal anthropometric data in a cohort of West African HIV+ adults .

**Methods:**

In a cohort of patients initiating antiretroviral treatment in Mali, we evaluated nutritional status using anthropometric measurements(weight, height, mid-upper arm circumference, waist circumference and triceps skinfold). Observers with no prior experience in the field of anthropometry were trained to perform anthropometrical measurements. To assess the intra- and inter-observer variability of the measurements taken in the course of the study, two sub-studies were carried out: one at the beginning and one at the end of the prospective study. Twelve patients were measured twice on two consecutive days by the same observer on both study occasions. The technical error of measurement (TEM) (absolute and relative value), and the coefficient of reliability (R) were calculated and compared across reliability studies.

**Results:**

According to the R and relative TEM, inter-observer reliabilities were only acceptable for height and weight. In terms of intra-observer precision, while the first and second anthropometrists demonstrated better reliability than the third, only height and weight measurements were reliable. Looking at total TEM, we observed that while measurements remained stable between studies for height and weight, circumferences and skinfolds lost precision from one occasion to the next.

**Conclusions:**

Height and weight were the most reliable measurements under the study's conditions. Circumferences and skinfolds demonstrated less reliability and lost precision over time, probably as a result of insufficient supervision over the entire length of the study. Our results underline the importance of a careful observer's selection, good initial preparation, as well as the necessity of ongoing training and supervision over the entire course of a longitudinal nutritional study. Failure to do so could have major repercussions on data reliability and jeopardize its utilization.

## Background

Malnutrition is an enduring preoccupation in sub-Saharan Africa. Despite its prevalence, few studies have examined its impact on disease progression and the treatment of prevailing health issues such as HIV. In this context, we have investigated the nutritional status of HIV+ patients initiating antiretroviral treatment (ART) in West Africa.

Anthropometric measurements are useful tools for the detection of deviations from normal nutritional status [1]. They also provide indications concerning lean body mass (LBM) and fat mass (FM). Variations of LBM and FM in quantities and distributions can be used as indicators of the global nutritional status [2]. Body mass index (BMI) (kg/m^2^) has been used as a marker of the global nutritional state, and chronic energy deficiency; and is useful for comparison across populations [3,4]. Mid-upper arm circumference (MUAC) is a better indicator of peripheral muscle and subcutaneous tissue wasting than BMI [5]. Its use has been proposed to replace or complement BMI in instances of humanitarian crises or emergencies [6]. Both markers have been associated with disease progression, risk of opportunistic infections and mortality before and after ART initiation among HIV patients [7-14]. Furthermore, since the advancement of ART, side effects affecting fat distribution from the limbs to the face, neck, back and abdominal regions such as lipodystrophy and lipoatrophy have been on the rise [15,16]. Anthropometric measurements indicative of lipid redistribution, such as skinfolds, hip and waist circumference, could be useful to document such manifestation.

### Reliability of anthropometric data

Despite offering many benefits (low costs, easy to perform, little equipment required), anthropometric techniques can be problematic due to their vulnerability to measurement errors and lack of reliability. Unreliability can be broken down into two components: 1) imprecision, referring to the measurement error variance due to intra- and inter-observer variability; and 2) undependability, a function of physiological variation, such as biological factors, that may influence the reproducibility of the measure [17,18]. Imprecision can arise from inadequate or improper training of personnel, difficulties in measurement of certain anthropometric characteristics such as skinfolds, and instrumental or technical errors. It remains the greater concern in anthropometry [19,20]. Imprecision can be especially problematic in large epidemiological studies that require multiple observers or that employ anthropometrists with little experience.

The technical error of measurement (TEM) is often employed to evaluate anthropometric measure imprecision. TEM is the standard deviation between repeated measurements taken independently by one observer (intra-observer) or between measurements performed by multiple anthropometrists (inter-observer) [21]. It uses the same units as the variable under consideration and can be employed in the calculations of confidence intervals [22]. In longitudinal studies, TEM may be used as an estimator of the proportion of the difference between two longitudinal measurements attributable to measurement error [18]. To facilitate the comparison of TEMs between anthropometric measurements or populations, conversion of absolute TEM to a relative TEM (%TEM) is often used.

Since TEM varies with age and with certain population characteristics, it can be difficult to determine acceptable levels. Alternatively, the reliability coefficient (R) can be used to compare anthropometric values in population studies [17]. R is the proportion of between-subject variance that is free from measurement error. It can be used to compare the relative reliability of different anthropometric methods between age groups [18]. The inter-observer reliability (R_inter_) and intra-observer reliability (R_intra_) can be calculated using TEM or %TEM. R and %TEM are related through the coefficient of variability (CV). R and %TEM thus illustrate different aspects of imprecision.

While anthropometric measurement errors have been examined in studies held in developed countries, the occurrence and extent of such errors have not always been systematically assessed in studies held in resource-limited countries. In such contexts, operational requirements for reliable data collection may be more difficult to meet, partly due to the scarcity of trained personnel and the greater challenges associated with data collection supervision.

Our objective was to evaluate the reliability of longitudinal anthropometric measurements collected in the context of a one-year cohort study of patients initiating ART in West Africa and to document the challenges associated with this measurement process. More specifically, we aimed at assessing measurement error of anthropometric data at the beginning and end of the cohort study, to evaluate the proportion of the longitudinal change that would be attributable to that error, and to determine whether reliability was adequate to allow anthropometric data usage in longitudinal analyses. Finally, we hoped to identify early indications of reliability issues that could benefit future longitudinal anthropometric studies held in similar contexts.

## Methods

### Study context

Two reliability studies were conducted in parallel to a one-year multi-centric cohort of 273 patients initiating ART in Mali. For each participating site, one observer was hired to collect data and perform anthropometric measurements. Observer 1 was a medical doctor with theoretical knowledge of anthropometry. Observers 2 and 3 had experience in conducting surveys, but none in anthropometry. All observers received initial training, which was supplemented with written instructions and practice every two weeks for the initial three months of the study.

### Study design

The two reliability studies were performed at three months (study A) and 18 months (study B) following cohort study initiation. On both occasions, 12 men and women were recruited from support groups for people living with HIV (PLWHIV). For each participant, weight, height, MUAC, triceps skinfold (TS), and waist circumference (WC) were measured by all three observers. For both reliability study, each participant was measured twice by each observer; once each day, on two consecutive days at approximately the same time of day. Heterogeneity of morphological traits among the participants was sought out. Volunteer characteristics were within the following ranges: age, 18-65; height, 152-186 cm; weight 41.5-99.4 kg; MUAC 21.3-40.6 cm; TS 4.50-64.67 mm; and WC, 63-110 cm. The variability observed among study participants fell within the range observed in cohort members.

Each observer was required to conduct and record his/her own measurements independently. Recorded measurement sets were kept concealed by each individual observer until the study end. The purpose and benefits of the study were explained to participants beforehand. They received monetary compensation for their participation. Confidentiality was maintained across the studies and analyses. Approval for this study was obtained from the ethical committees of the National Institute of Public Health Research in Mali and the Montreal University Hospital Center (CHUM).

### Anthropometric measurements

Body weight was measured in kilograms accurate to the closest gram using an eye-level mechanical balance beam with sliding counterweights *(Detecto)*. Height was measured to the nearest 0.5 cm using the scale's stadiometer. The same scale and stadiometer were used by all observers. The scale was calibrated at the beginning of the day before initiating measurements. TS were measured to the nearest 0.5 mm on the right side of the body using a *Slim Guide Skinfold *caliper. Skinfold measurement was repeated three times and averaged for error estimation. MUAC was measured at the mid-point between the uppermost edge of the posterior border of the acromion process and the tip of the olecranon process. A mark was made on the skin at this position and circumference was measured horizontally. WC was measured at the level of the uppermost limits of the ileum. Marks were made on the skin at these locations and circumference was measured horizontally. Both circumferences were calculated to the closest 1 mm using a non stretchable, flexible vinyl *Gulick *measuring tape. The tape was spring loaded to offer a high level of accuracy with consistent tension. Neither the tapes, calipers, nor stadiometer were calibrated.

### Statistical analysis

TEM is commonly used to evaluate the imprecision of measurements taken by different observers on the same subject (inter-observer error) or between repeated measures performed on different occasions of the same subject by the same observer (intra-observer error) [17,18] (*see appendix I for equations*). As indicated in the literature, acceptable TEM values should be of the order of 0.1 kg for weight, 3 mm for height and 2 mm for girth (limbs) [23]. Using the best and worse TEM per observer or study occasion we calculated 95% confidence intervals (CI) [18,23,24].

To compare TEM across anthropometric measurements or study occasions, we converted the absolute TEM to %TEM[21]. Acceptable %TEM levels were 5% or less for skinfolds, and 1% for other anthropometrical measures [25]. While %TEM allows for comparison of different anthropometric measures, it provides no information for comparison between studies using more than two observers or in which intra- and inter-observer TEM are calculated [18]. Total TEM is preferred in those instances. Finally, when looking at R, R values > 0.95 were sought [26].

Independent-samples t-test was used to compare study populations. Calculations were done with Excel 2003 and SPSS 17.0.

## Results

On both study occasions (A and B), 12 adult subjects were recruited through support groups for people living with HIV. However in study B, one of the participants did not return on the second day and analyses had to be restricted to the 11 returning patients. No significant differences were found between the two study populations except for MUAC and WC variance which was smaller in study B. For cultural reasons, it was not feasible to measure hip circumference and, in some instances, WC, as patients felt uncomfortable about exposing those areas. Consequently, WC analyses in study B only included seven patients. Finally, comparisons of anthropometric characteristics between participants in our reliability and cohort studies indicated that the former had slightly, but significantly, higher weight, arm and hip circumferences compared to our cohort participants (*data not shown*).

### Inter observer reliability

The coefficient of variability, inter-observer absolute and relative TEM as well reliability coefficients for each anthropometric measurement on both study occasions are shown in Table 1. Based on accepted error standards, %TEM for weight and height were considered acceptable in most instances. This was not the case for MUAC and WC %TEM which were frequently between 2.5 and 3%. As for skinfolds, levels of %TEM were more than 10 times above the acceptable standards indicating very poor reliability.

**Table 1:** Inter-observer TEM, %TEM and reliability coefficient by study occasion and anthropometric measure

	CV	TEM	%TEM	R
	
Study A				
Height				
*Day 1*	0.05	0.90	0.53	0.98
*Day 2*	0.05	1.64	0.98	0.96
Weight				
*Day 1*	0.23	0.97	1.37	0.99
*Day 2*	0.23	0.30	0.43	0.99
MUAC				
*Day 1*	0.17	0.73	2.53	0.98
*Day 2*	0.17	0.43	1.48	0.99
TS				
*Day 1*	0.64	17.6	83.7	0.00
*Day 2*	0.62	4.61	18.8	0.91
WC				
*Day 1*	0.14	2.05	2.44	0.97
*Day 2*	0.14	2.34	2.78	0.96
**Study B**				

Height				
*Day 1*	0.05	0.53	0.32	0.99
*Day 2*	0.05	1.91	1.15	0.95
Weight				
*Day 1*	0.20	0.50	0.76	0.99
*Day 2*	0.20	0.42	0.62	0.99
MUAC				
*Day 1*	0.12	1.30	4.59	0.84
*Day 2*	0.10	0.76	2.69	0.93
TS				
*Day 1*	0.62	14.3	62.3	0.00
*Day 2*	0.59	14.4	58.2	0.00
WC				
*Day 1*	0.07	2.29	2.83	0.82
*Day 2*	0.06	1.98	2.33	0.86

TEM: Technical error of measurement

CV: Coefficient of variability

MUAC: Mid-upper arm circumference

TS: Triceps skinfold

According to the reliability coefficient, height and weight inter-observer variability were acceptable in both studies. However, MUAC and WC inter-observer reliability went from being acceptable in the first study to unacceptable in the second study. Finally, TS precision was poor at all times.

There was no general trend in the absolute or relative TEM between study A and study B, or between the first and the second day of each study. However, drops of the reliability coefficient below the 0.95 cutoff were more frequent in the second study, especially for circumference measurements. Indeed, while MUAC and WC appeared reliable, according to R, in study A; they both had lost precision at the time of study B.

### Intra observer reliability

Analysis of intra-observer %TEM showed that only height and weight met acceptability standards in some instances (Table 2). This was not the case for circumference and skinfold measurements which did not demonstrate acceptable reliability at any time. Similar observations were made about R. However, as observed in Table 1, there was a diminution in precision, based on R, at the time of study B. This was especially true for TS and WC.

**Table 2:** Intra-observer TEM, %TEM and reliability coefficient by study occasion and anthropometric measure

	CV	TEM	%TEM	R
	
Observer 1				
Height				
*Study A*	0.04	0.62	0.37	0.99
*Study B*	0.05	0.53	0.32	0.99
Weight				
*Study A*	0.23	0.84	1.18	0.99
*Study B*	0.19	0.66	0.99	0.99
MUAC				
*Study A*	0.18	0.35	1.21	0.99
*Study B*	0.11	1.66	5.78	0.73
TS				
*Study A*	0.64	4.06	16.0	0.94
*Study B*	0.40	1.09	10.2	0.94
WC				
*Study A*	0.14	1.22	1.46	0.99
*Study B*	0.08	1.64	1.95	0.94
**Observer 2**				

Height				
*Study A*	0.05	0.90	0.53	0.99
*Study B*	0.05	0.52	0.32	0.99
Weight				
*Study A*	0.23	0.51	0.71	0.99
*Study B*	0.20	0.58	0.87	0.99
MUAC				
*Study A*	0.17	0.51	1.77	0.99
*Study B*	0.11	0.36	1.27	0.99
TS				
*Study A*	0.59	2.65	10.6	0.97
*Study B*	0.60	4.67	17.2	0.92
WC				
*Study A*	0.14	2.01	2.41	0.97
*Study B*	0.09	3.33	4.08	0.79
**Observer 3**				

Height				
*Study A*	0.05	2.07	1.22	0.94
*Study B*	0.05	1.00	0.60	0.97
Weight				
*Study A*	0.23	0.65	0.93	0.99
*Study B*	0.20	2.26	3.36	0.97
MUAC				
*Study A*	0.17	0.49	1.71	0.99
*Study B*	0.11	0.69	2.45	0.95
TS				
*Study A*	0.63	4.92	22.2	0.88
*Study B*	0.26	2.75	8.22	0.90
WC				
*Study A*	0.15	1.45	1.71	0.99
*Study B*	0.06	2.04	2.49	0.80

TEM: Technical error of measurement

CV: Coefficient of variability

MUAC: Mid-upper arm circumference

TS: Triceps skinfold

WC: Waist circumference

Furthermore, observer 3 performed rather poorly on almost every anthropometric measurement and on both study occasions as indicated by the relative TEM and reliability coefficient. This is indicative of the observer's lack of consistency when executing the measurements. Overall, observer 2 appeared to be the most precise.

### Total variability between sub-studies

Based on % total TEM, our results indicated that height was the only reliable measurement, which held true on both study occasions (Table 3).

**Table 3:** Comparison of total TEM between studies*

	Total TEM	% Total TEM	R
Height			
*Study A*	1.62	0.96	0.96
*Study B*	0.89	0.54	0.99

Weight			
*Study A*	1.18	1.68	0.99
*Study B*	1.49	2.24	0.99

MUAC			
*Study A*	0.86	2.98	0.97
*Study B*	1.67	5.93	0.74

TS			
*Study A*	18.1	85.9	0
*Study B*	14.6	63.8	0

WC			
*Study A*	2.05	3.10	0.95
*Study B*	3.35	4.14	0.61

* Results are presented only for the first day of each study

When examining reliability coefficients, we observed that R remained above the 0.95 cutoff for height and weight in both studies. This was not the case for MUAC and WC. For these measurements, reliability, as indicated by R, was considered acceptable in the initial study. There was, however, a noticeable drop in precision at the time of study B. In the course of the second study, % total TEM notably increased and R decreased dramatically especially for MUAC and WC.

Total TEM of TS was quite poor in both study occasions indicating mediocre precision of that variable as measured in our study.

### TEM utility and implication for the cohort study

As indicated by TEM fluctuations between studies, measurement error varies through time. In longitudinal studies such as our cohort study, knowledge of TEM can be used to evaluate whether the difference between two longitudinal measurements is a true difference or an artifact resulting from measurement error. We used the best and worst TEM per observer or study occasion to evaluate the proportion of the difference between two measures that could be attributable to measurement error (Table 4). To do so, six-month weight and MUAC gain reported in similar cohorts were used [27,28].

**Table 4:** 95% confidence intervals for imprecision based on previously reported 6-month weight and MUAC gain

			Best case		Worst case	
			
	6-month gain		TEM	% gain represented by ± 2TEM	TEM	% gain represented by ± 2TEM
	
Weight (kg)	2.8*					
		Obs. 1 (intra)	0.66	65.3	0.84	83.2
		Obs. 2 (intra)	0.51	50.5	0.58	57.4
		Obs. 3 (intra)	0.65	64.3	2.26	**223.7**
		Inter	0.30	29.7	0.97	96.0
		Total^‡^	0.68	67.3	1.74	**172.1**
**MUAC (cm)**	1†					

		Obs. 1 (intra)	0.35	97.0	1.66	**460.1**
		Obs. 2 (intra)	0.36	99.8	0.51	**141.4**
		Obs. 3 (intra)	0.49	**135.8**	0.69	**191.3**
		Inter	0.43	**119.2**	1.30	**360.3**
		Total^‡^	0.77	**213.6**	1.57	**435.1**

* Obtained from Saghayam *et al*. (2007)

† Obtained from Kamya *et al. *(2007)

‡ Total TEM calculated using best or worse intra- and inter-TEM as indicated in table 4

In the best case, 50.5 to 65% of a 2.8 kg six-month weight gain could be attributable to intra-observer error if it had been observed in our studies. In the worse case, up to 223.7% (observer 3) could have been interpreted as being attributable to measurement error (Table 4). As for MUAC, even in the best case scenario, between 97 and 135.8% of the six-month gain could have been the result of intra-observer measurement error. Overall, a six-month weight gain of 2.8 kg would have been measured without undue imprecision only if measurements had been performed by observer 1 or 2. If similar weight increments had been observed in our study, we would have had to conclude that, at best 67.3% of that gain could be attributable to total TEM. In the worse scenario, six-month weight gain could have been interpreted as consisting at 172.1% of total TEM.

## Discussion

Three main observations could be drawn from our data. First, height and weight were the only reliable anthropometric measures either from an intra- or inter-observer perspective. Second, MUAC and WC were mostly imprecise while skinfolds demonstrated very poor reliability independently of the imprecision measure used. Third, we observed a reduction of the overall reliability of all measures between the first and second study.

Comparison of our results to previously published TEM and R values indicated that while weight, MUAC and WC fell within the range of formerly reported inter-observer values, height and TS did not [18]. Both variables have been measured with greater imprecision in our studies than in earlier investigations. Evaluation of intra-observer TEM similitude to previous reports indicated that our observers performed very variably in comparison to previous reports. Weight was the only measure for which all of our observers' TEM fell outside the range of previously reported intra-observer error values [18]. This was somewhat surprising considering that weight seemed to be the most reliable variable. Comparison of total TEM data to maximum reference values reported in the literature indicated that weight was the only measurement for which our results remained below the maximum acceptable TEM on both study occasions; height was only acceptable in study B. Total TEMs for all other measurements were above the suggested maximum acceptable total TEM[26].

The unreliability of the data collected in the course of our investigation parallels results obtained in similar circumstances (large epidemiologic studies employing recently trained anthropometrists with limited experience)[18]. However these studies were conducted in developed countries. Moreover, the lesser vulnerability of weight and height to imprecision, as observed here, has been frequently documented [29]. Those measures implicate less subjective appreciation than that of circumferences and skinfold; the reliability of the later being often problematic in large epidemiological study [18].

As reported by Ross et al. (1994), we noticed considerable variability in the R-%TEM relationship[30]. In that, a lower %TEM was not consistently associated with a higher R. Indeed, in some instances we noticed that a low %TEM was associated with a high reliability coefficient (R > 0.95). This could be due to R being a function of the measure's CV. It suggests that when working with a more homogenous study population, a high R can be associated with a smaller %TEM. A reverse association would be observed when dealing with greater heterogeneity as illustrated in Table 2. In looking at the CV of WC, we observed a decrease between the first and second study by almost half. Consequently, for similar errors of measurement, R was deemed inacceptable in study B but not in study A.

### Limitations and challenges

As mentioned by many authors, standardization, training in anthropometric measurement and regular quality control are important prerequisites to insure quality and reliability of the data [29]. In resource-limited settings, these requirements may be more difficult to meet [31]. Indeed, in our cohort study, anthropometric assessment requirements, such as observer training and data collection supervision, were revealed to be more challenging to implement than first conceptualized. First, due to contextual and logistical limitations, it was impossible to find a nutritionist with experience in anthropometry to act as a reference and vigilant, or to hire trained observers in anthropometry. Second, though the training given to the observers was quite intensive before cohort initiation and repeated every two weeks during the first three months of the cohort study (at the end of which we held study A), it could not be maintained thereafter. Study B was conducted after 15 months of drought in terms of training and data collection supervision; a gap which probably contributed to the decrease in precision. Lastly, early data had indicated reliability issues with observer 3 and concerns about the observer's capacity to perform at the job at hand. These warnings should have been better taken into account as it later revealed impossible, for political and legal reasons, to replace the observer.

Unreliability may have arisen from the tools used to perform anthropometry. More complex instruments such as calipers, are associated with greater equipment bias than tapes, for example. However, the degree of inaccuracy resulting from these instruments was not assessed in the course of our study. While the same brand of instruments were used by each observer, the wear-and-tear that could have affected the precision of each tool (especially calipers) was probably uneven [32,33]. Furthermore, differences in degree of compression and size of measurement are known to vary between calipers from the same manufacturer [18]; differences that we did not assess. However, the type of balance, stadiometer and measuring tape used in our study were standard and required little or no calibration. We believe that little variability could have originated from these instruments. On the other hand, more accurate calipers such as *Lange *could have been preferred. For these reasons, generalizability of the conclusions concerning skinfolds may be limited to studies using *Slim Guide *calipers.

Finally, errors in anthropometry can also be attributed to alterations in the composition and physical properties of tissues [29]. It is possible, for example, that variations in the state of hydration and nourishment may have occurred between study days and modified certain parameters such as weight. However, those variations were probably minute since weight measurements appeared to be reliable in our study.

Our results clearly indicated reliability issues with MUAC, WC and TS which will limit their utilization in longitudinal analyses. The lack of a true reference measure, for comparison purposes, will not allow adjustment for the errors in exposure assessments [34-36]. However, utilization of biased measures could lead to a patient's misclassification into the wrong exposure category, leading authors to draw erroneous conclusions.

Finally, while the sample size used was small and heterogeneous, it was nonetheless almost identical to that used in the methodology used in previous studies [18,29,37].

## Conclusion

Our results indicate that height and weight are the only measures sufficiently reliable to be used in future analyses in this study. The reliability of these measurements, and indirectly of BMI, is reassuring since BMI is a useful tool to detect chronic energy deficiency[4,38] and has also been proposed as an indicator of HIV progression in developing countries [14]. Our data suggest that the value of this prognostic tool would probably be reliable even when employing observers with little experience, as reported here.

On the other hand, the unreliability of MUAC and WC would probably lead to a misclassification bias and erroneous conclusions if used in further analyses. This is unfortunate since MUAC can be a useful tool to detect malnutrition under certain circumstances[6]. MUAC measurement requires little material and no calibration is necessary, making it ideal for nutritional assessment in remote regions. MUAC can be used as a proxy of BMI and may be a better indicator of lean body mass depletion[5]. However, as indicated here, the greater requirement for sustained training (compared with the measurement of height and weight) would jeopardize its usefulness in a context where trained supervisors and constant training are not accessible.

Although skinfolds may be considered by some authors as a good field technique[39] and best at estimating body fat[40], they are also recognized for their high vulnerability to imprecision, as demonstrated here. Consequently, would only recommend their use if continuous training and evaluation opportunities are available

Looking at the decline in reliability across studies (A and B), we can imagine that it could have been prevented by ongoing training between the studies. We thus strongly recommend that the following key elements be met to insure successful and reliable data collection: 1) Researchers should select and screen out observers carefully before study initiation to insure their capacity to follow protocols and execute the task at hand. It might later reveal itself as difficult to fire an unsuitable observer. Although this goes beyond the scope of this paper, we suggest investigating national employment laws before hiring local observers; 2) Observers should receive intensive initial training with an early evaluation of reliability and performance of anthropometric measurements. This preliminary phase should be followed by frequent updates, calibration checkups, combined with measurement reliability assessments (comparing the observer's measurement to that of a nutritionist acting as a gold standard) during the entire course of the study; and 3) Data collection should be carefully supervised throughout the entire length of the study. The availability of an 'expert' in anthropometry may be crucial not only during the training process but also during data collection supervision. By comparing the data gathered to that of a gold standard, it could be possible to 'calibrate' for the bias due to errors in exposure measurement [34]. We would like to stress that the difficulties we encountered in terms of staff training and execution of correct measurements are independent of the contextual setting. Similar problems are commonly encountered in high-income settings, but resources are more readily available in such contexts.

## Abbreviations

ART: (Antiretroviral treatment); BMI: (Body mass index); CI: (Confidence interval); HIV: (Human immunodeficiency virus); LBM: (Lean body mass); FM: (Fat mass); MUAC: (Mid-upper arm circumference); PLWHIV: (People living with HIV); TEM: (Technical error of measurements); TS: (Triceps skinfold); WC: (Waist circumference).

## Competing interests

The authors declare that they have no competing interests.

## Authors' contributions

MS wrote the paper, performed the analyses and interpreted the results. ML and MVZ contributed to the interpretation of results and paper revision. SAA co-ordinated the reliability and cohort studies. VKN revised the paper and initialized the cohort study. All authors have seen and approved the final version of the manuscript.

## Appendix

### Equations

When evaluating the reliability of two measurements (whether two measures from the same observer or one measure from two different observers) equation 1 was used, where D represents the difference between the two measurements and N the number of individuals measured [18].

(1) $$\text{TEM}=\surd (\Sigma {\text{D}}^{\text{2}})/\text{2N}$$

When more than two observers were involved, equation 2 was used where K is the number of observers (one determination per observer) and M is the measurement value (equation2) [18].

(2) $$\text{TEM}=\surd ((\Sigma^{\text{N}}((\Sigma^{\text{K}}{\text{M}}^{\text{2}})-({\left(\Sigma^{\text{K}}\text{M}\right)}^{\text{2}}/\text{K})))/\text{N}(\text{K}-\text{1}))$$

Using the best and worst TEM per observer or study occasion we calculated 95% confidence intervals (CI). To do so, equation 3 was applied [18,23,24] :

(3) $$\text{95}\%\text{CI}=\text{1}.\text{96}\surd {\left(\text{TEM}\right)}^{\text{2}}+{\left(\text{TEM}\right)}^{\text{2}}$$

To converted an absolute TEM to a relative TEM (%TEM), we used the equation proposed by Norton & Old (1996) [21].

(4) %TEM=TEM/mean×100

Total TEM (equation 5) where TEM (intra_1_) is the intra-observer TEM for the first observer[18]:

(5) $$\begin{array}{c} {\text{Total TEM}}_{\left(\text{for 3 observers}\right)}=\surd ((\text{TEM }({\left({\text{intra}}_{\text{1}}\right)}^{\text{2}}+\text{TEM }({\left({\text{intra}}_{\text{2}}\right)}^{\text{2}}\\ +\text{ TEM }\left({\left({\text{intra}}_{\text{3}}\right)}^{\text{2}}\right)/\text{3}+\text{TEM }{\left(\text{inter}\right)}^{\text{2}} \end{array}$$

The coefficient of reliability (R) was calculated using equation 6, where mean refers to the measurement's average and SD refers to the standard deviation for that measurement.

(6) $$\text{R}=\text{1}–({\text{TEM}}^{\text{2}}/{\text{mean}}^{\text{2}})/({\text{SD}}^{\text{2}}/{\text{mean}}^{\text{2}})$$

## Pre-publication history

The pre-publication history for this paper can be accessed here:

http://www.biomedcentral.com/1471-2288/10/102/prepub
